# Supplementary figures and images for: The population affected by dust in China in the springtime
Source: PLoS One. 2024 Feb 23;19(2):e0281311. doi: 10.1371/journal.pone.0281311 (PMC10889670; doi:10.1371/journal.pone.0281311)

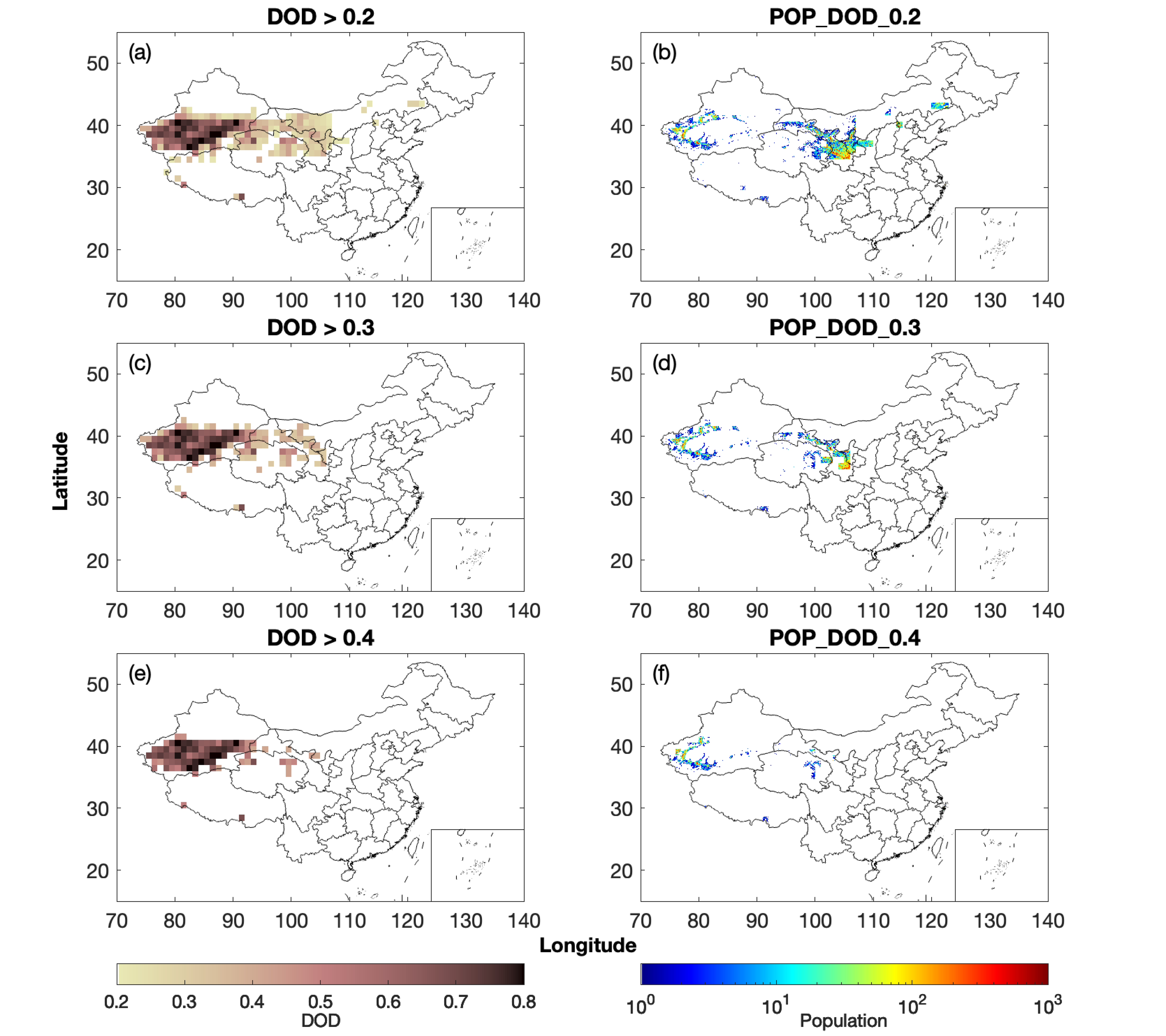

Supplement: S1 Fig — Locations with March-April mean DOD above (a) 0.2, (c) 0.3, and (e) 0.4 in 2003 using MODIS and the spatial distribution of the population affected by DOD above (b) 0.2, (d) 0.3, and (f) 0.4 in 2003. (TIF) [file pone.0281311.s001.tif]

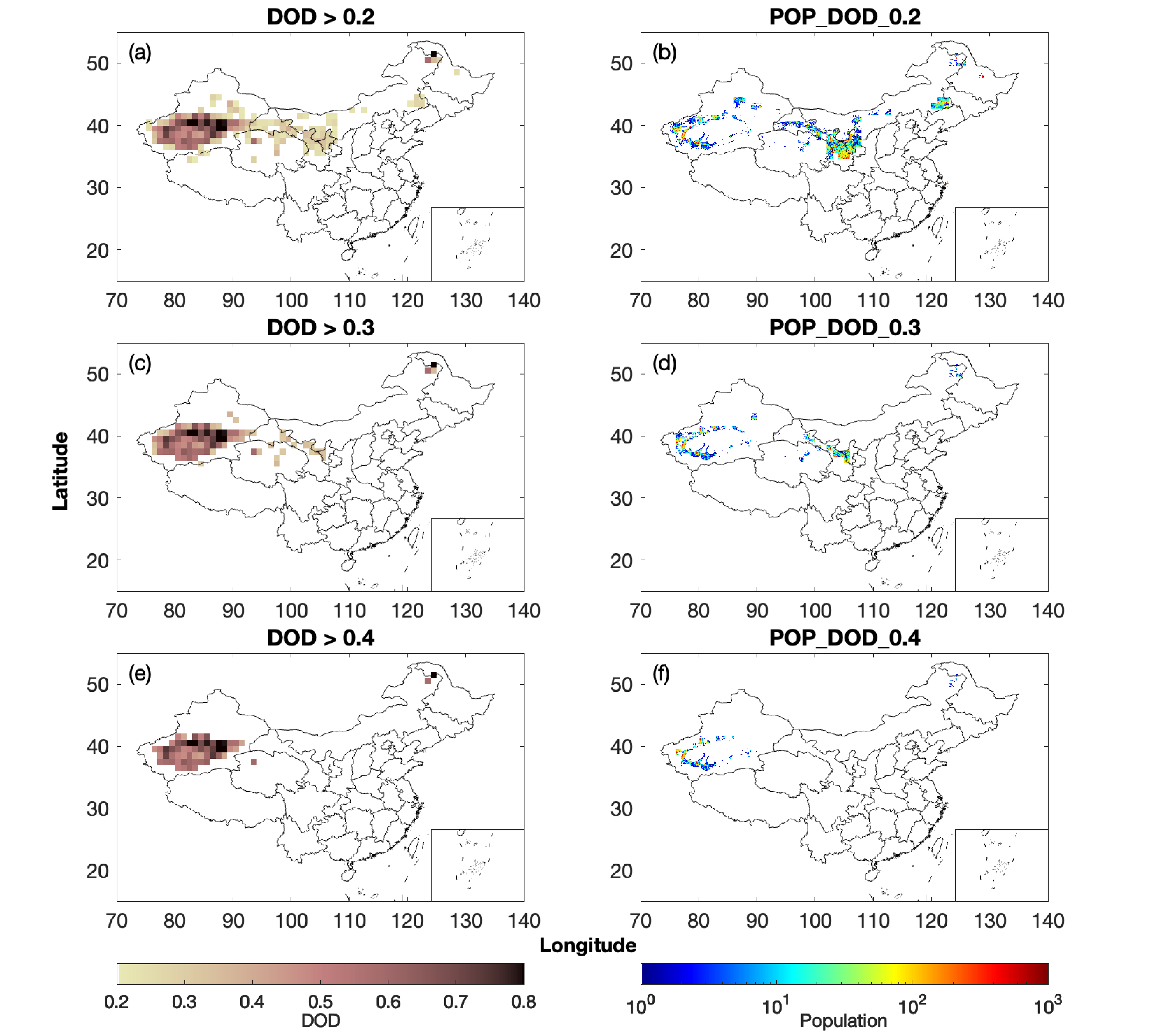

Supplement: S2 Fig — Locations with March-April mean DOD above (a) 0.2, (c) 0.3, and (e) 0.4 in 2004 using MODIS and the spatial distribution of the population affected by DOD above (b) 0.2, (d) 0.3, and (f) 0.4 in 2004. (TIF) [file pone.0281311.s002.tif]

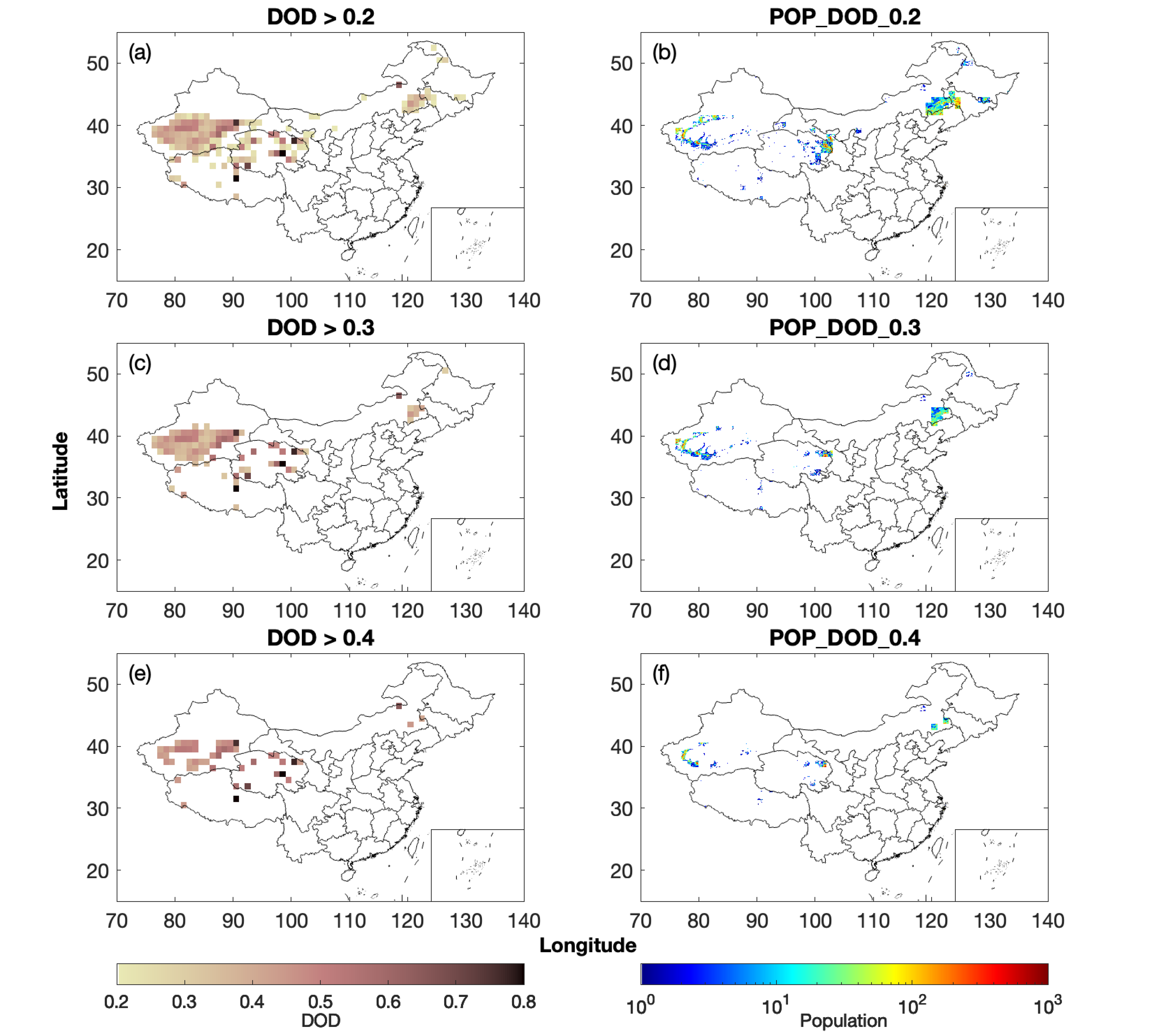

Supplement: S3 Fig — Locations with March-April mean DOD above (a) 0.2, (c) 0.3, and (e) 0.4 in 2005 using MODIS and the spatial distribution of the population affected by DOD above (b) 0.2, (d) 0.3, and (f) 0.4 in 2005. (TIF) [file pone.0281311.s003.tif]

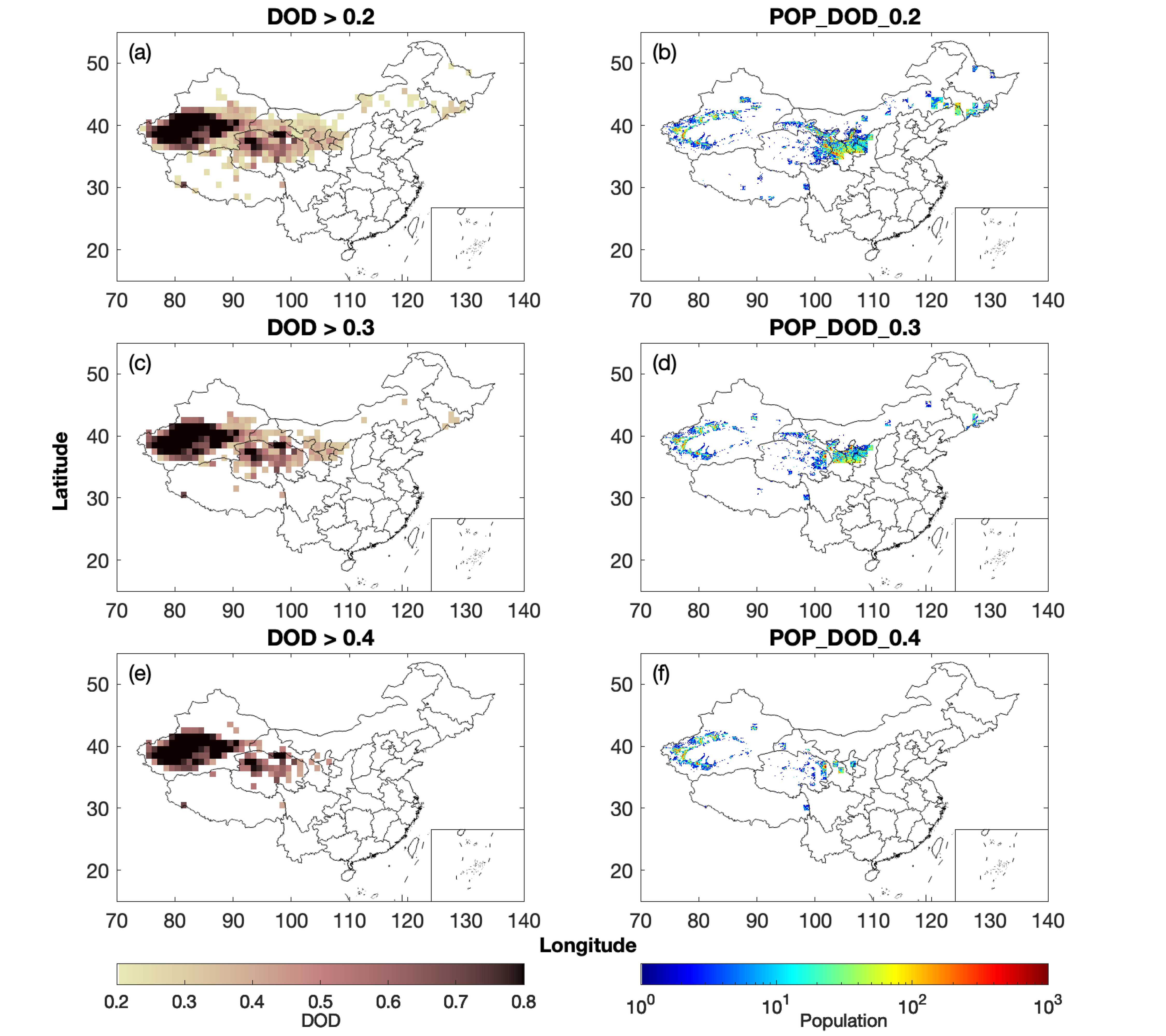

Supplement: S4 Fig — Locations with March-April mean DOD above (a) 0.2, (c) 0.3, and (e) 0.4 in 2007 using MODIS and the spatial distribution of the population affected by DOD above (b) 0.2, (d) 0.3, and (f) 0.4 in 2007. (TIF) [file pone.0281311.s004.tif]

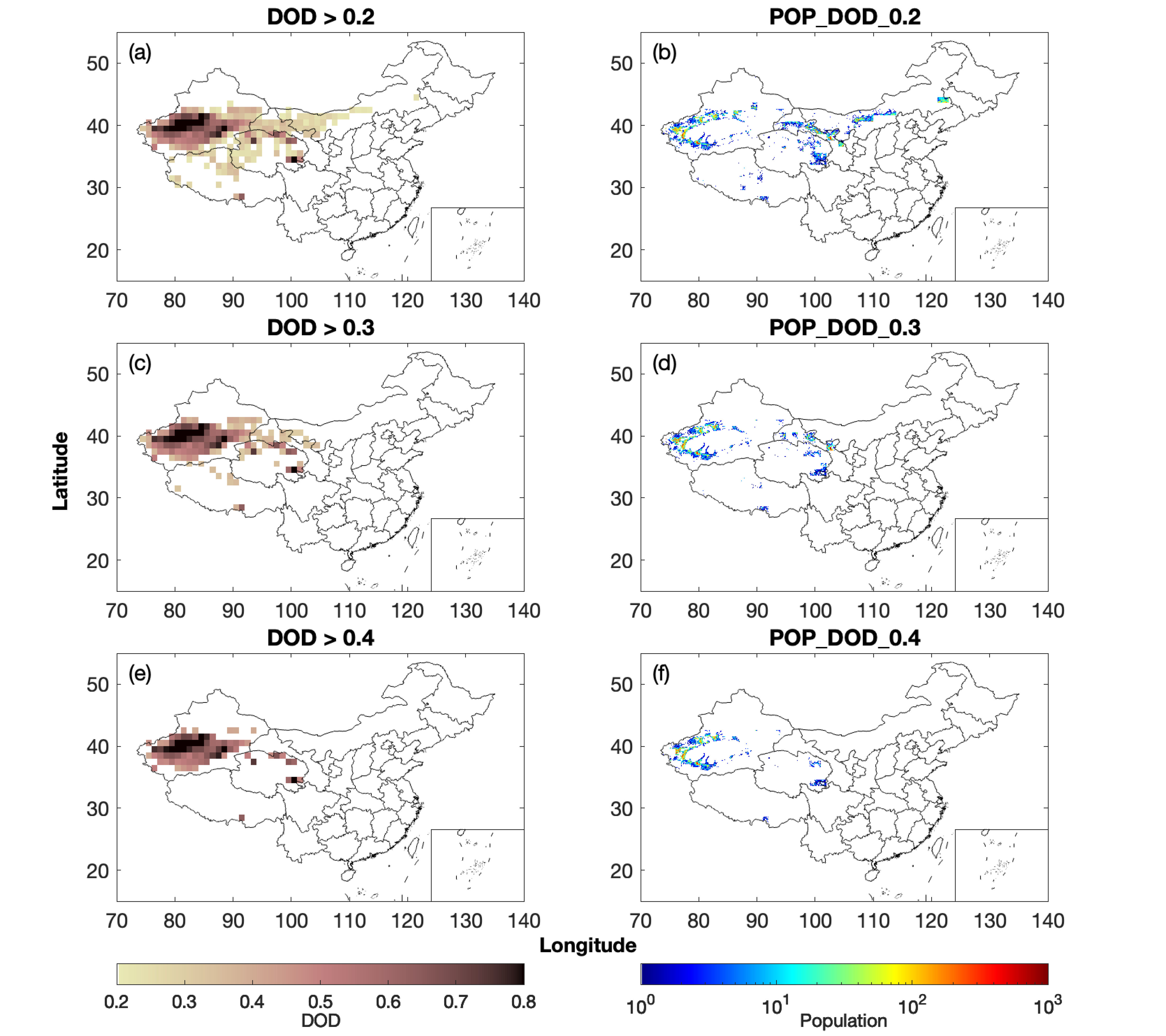

Supplement: S5 Fig — Locations with March-April mean DOD above (a) 0.2, (c) 0.3, and (e) 0.4 in 2008 using MODIS and the spatial distribution of the population affected by DOD above (b) 0.2, (d) 0.3, and (f) 0.4 in 2008. (TIF) [file pone.0281311.s005.tif]

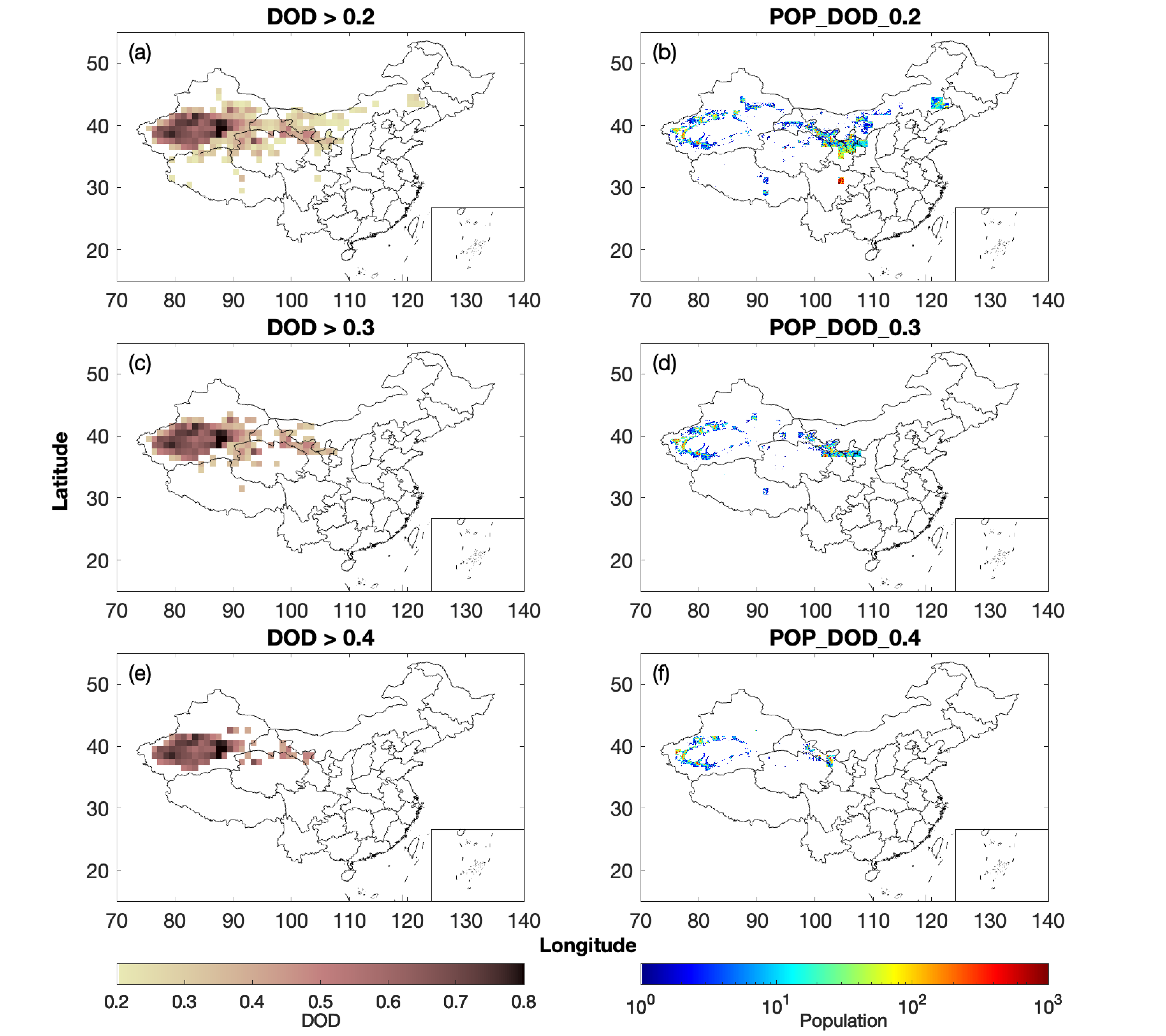

Supplement: S6 Fig — Locations with March-April mean DOD above (a) 0.2, (c) 0.3, and (e) 0.4 in 2009 using MODIS and the spatial distribution of the population affected by DOD above (b) 0.2, (d) 0.3, and (f) 0.4 in 2009. (TIF) [file pone.0281311.s006.tif]

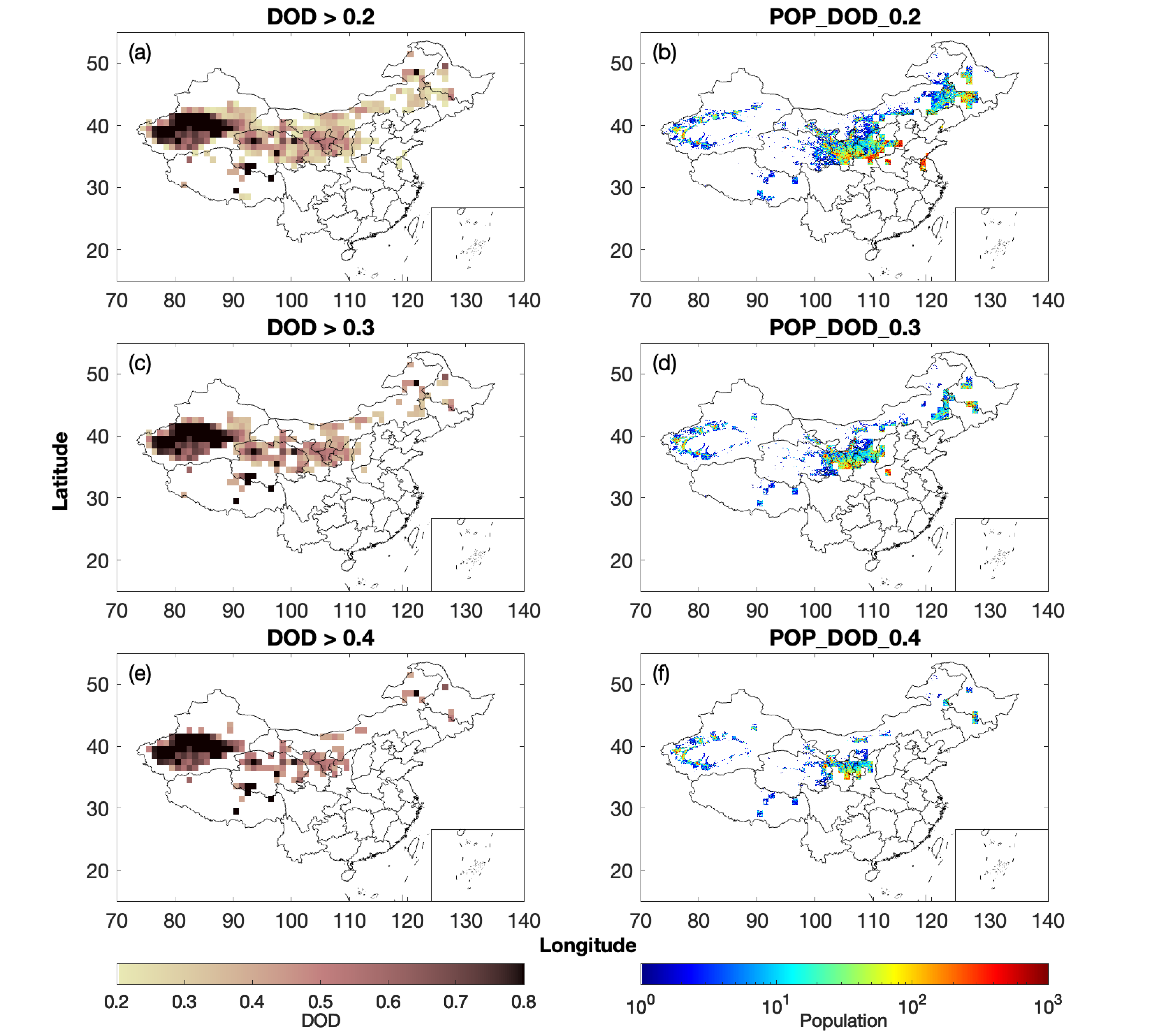

Supplement: S7 Fig — Locations with March-April mean DOD above (a) 0.2, (c) 0.3, and (e) 0.4 in 2010 using MODIS and the spatial distribution of the population affected by DOD above (b) 0.2, (d) 0.3, and (f) 0.4 in 2010. (TIF) [file pone.0281311.s007.tif]

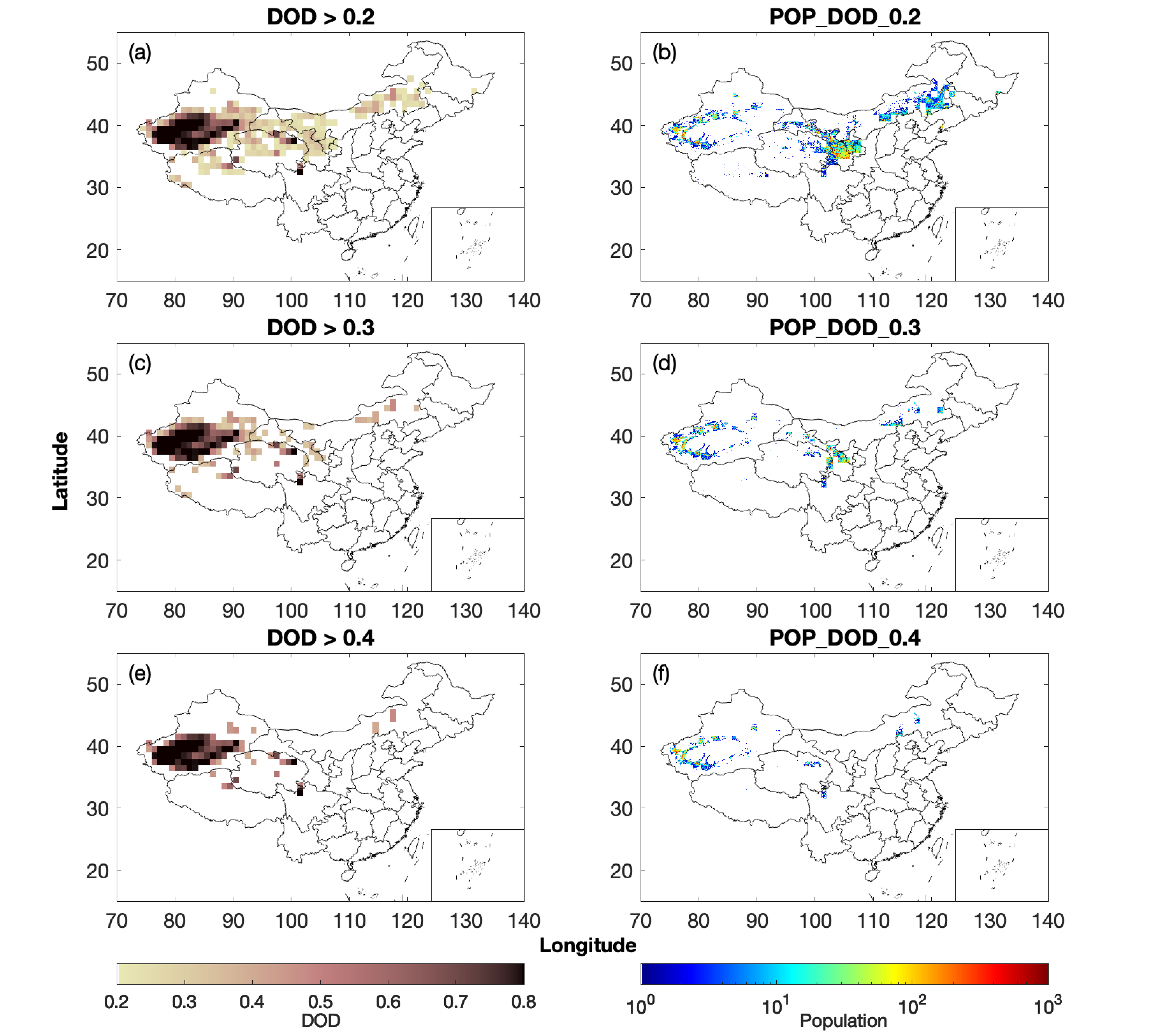

Supplement: S8 Fig — Locations with March-April mean DOD above (a) 0.2, (c) 0.3, and (e) 0.4 in 2011 using MODIS and the spatial distribution of the population affected by DOD above (b) 0.2, (d) 0.3, and (f) 0.4 in 2011. (TIF) [file pone.0281311.s008.tif]

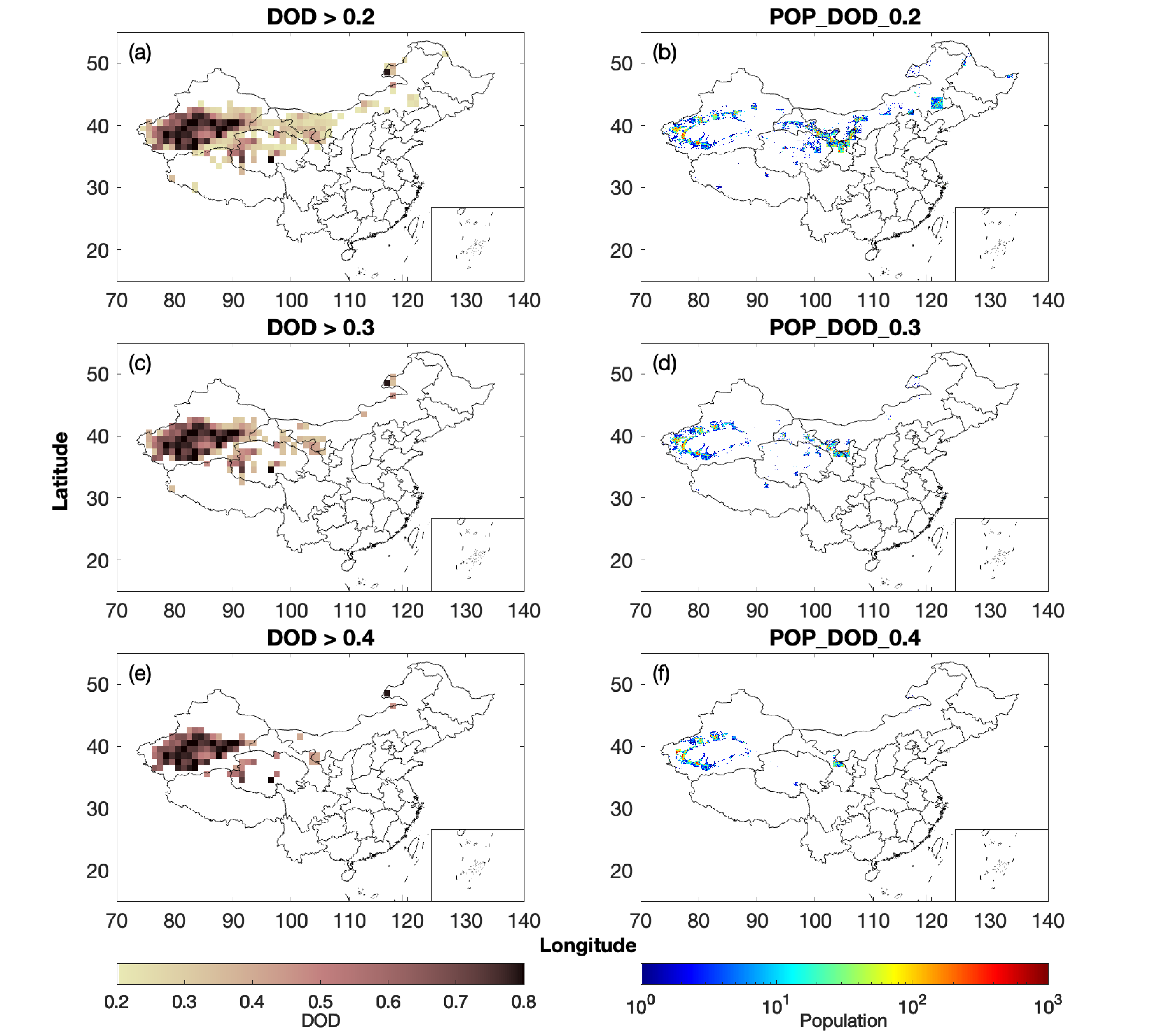

Supplement: S9 Fig — Locations with March-April mean DOD above (a) 0.2, (c) 0.3, and (e) 0.4 in 2012 using MODIS and the spatial distribution of the population affected by DOD above (b) 0.2, (d) 0.3, and (f) 0.4 in 2012. (TIF) [file pone.0281311.s009.tif]

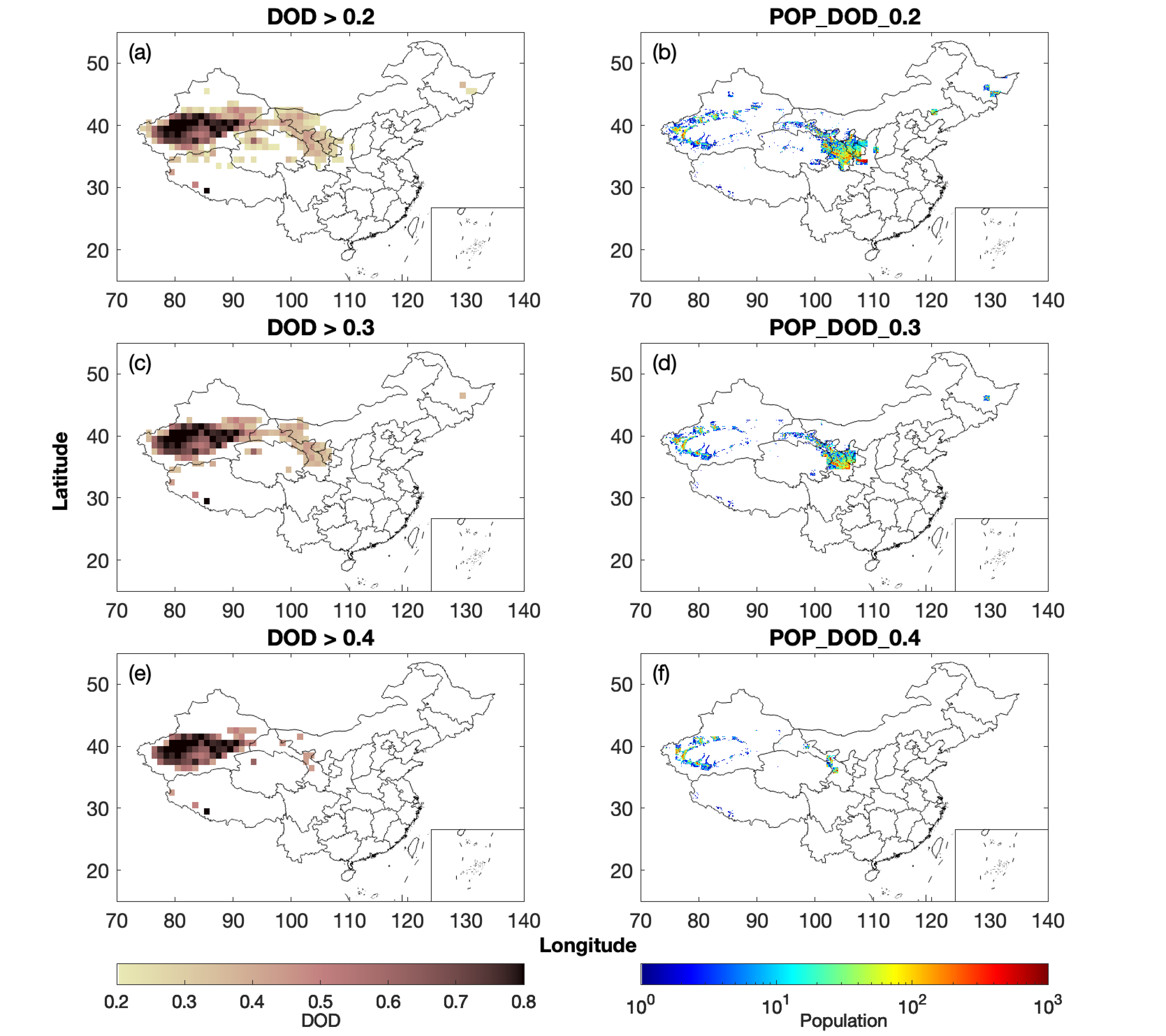

Supplement: S10 Fig — Locations with March-April mean DOD above (a) 0.2, (c) 0.3, and (e) 0.4 in 2013 using MODIS and the spatial distribution of the population affected by DOD above (b) 0.2, (d) 0.3, and (f) 0.4 in 2013. (TIF) [file pone.0281311.s010.tif]

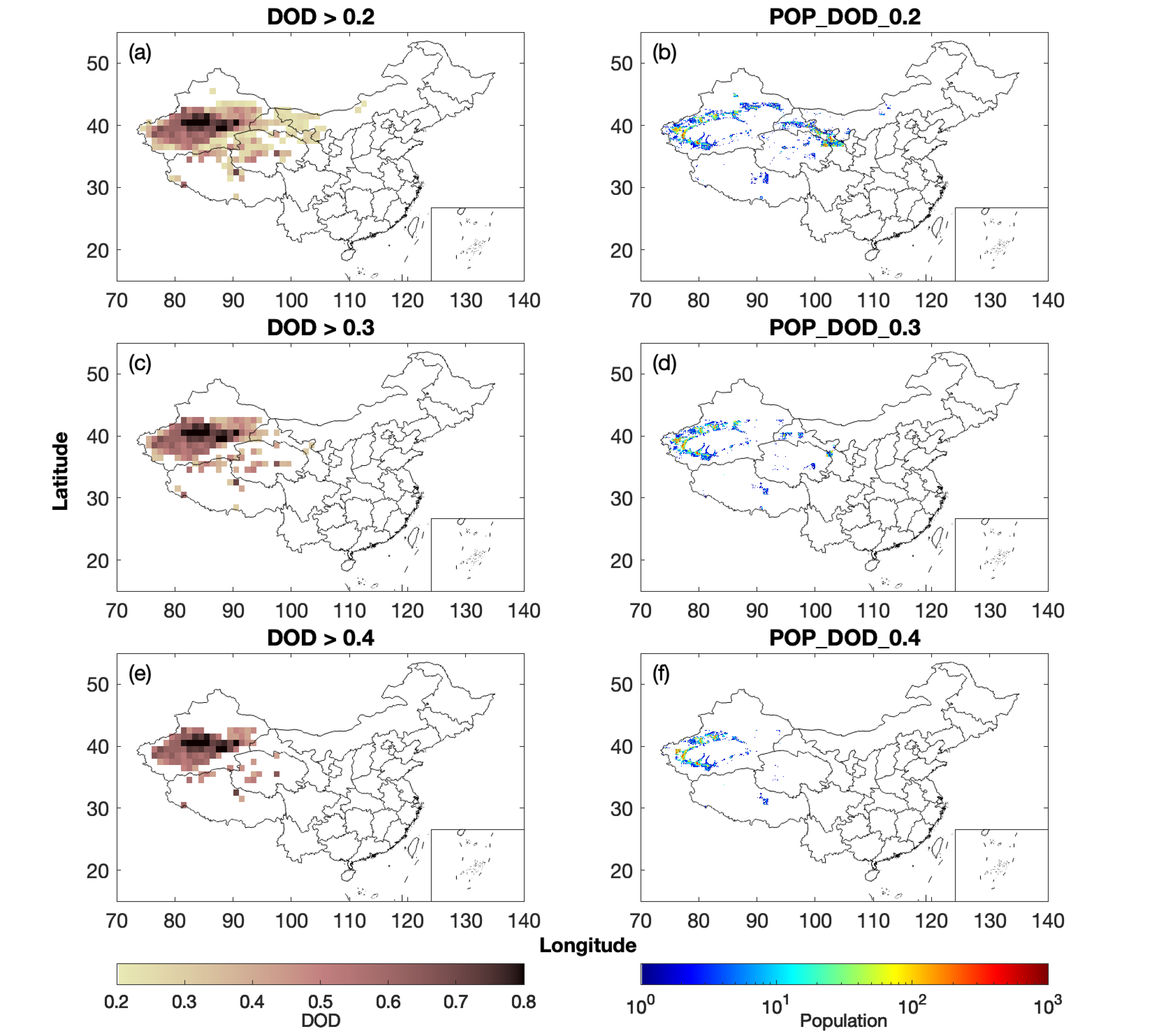

Supplement: S11 Fig — Locations with March-April mean DOD above (a) 0.2, (c) 0.3, and (e) 0.4 in 2014 using MODIS and the spatial distribution of the population affected by DOD above (b) 0.2, (d) 0.3, and (f) 0.4 in 2014. (TIF) [file pone.0281311.s011.tif]

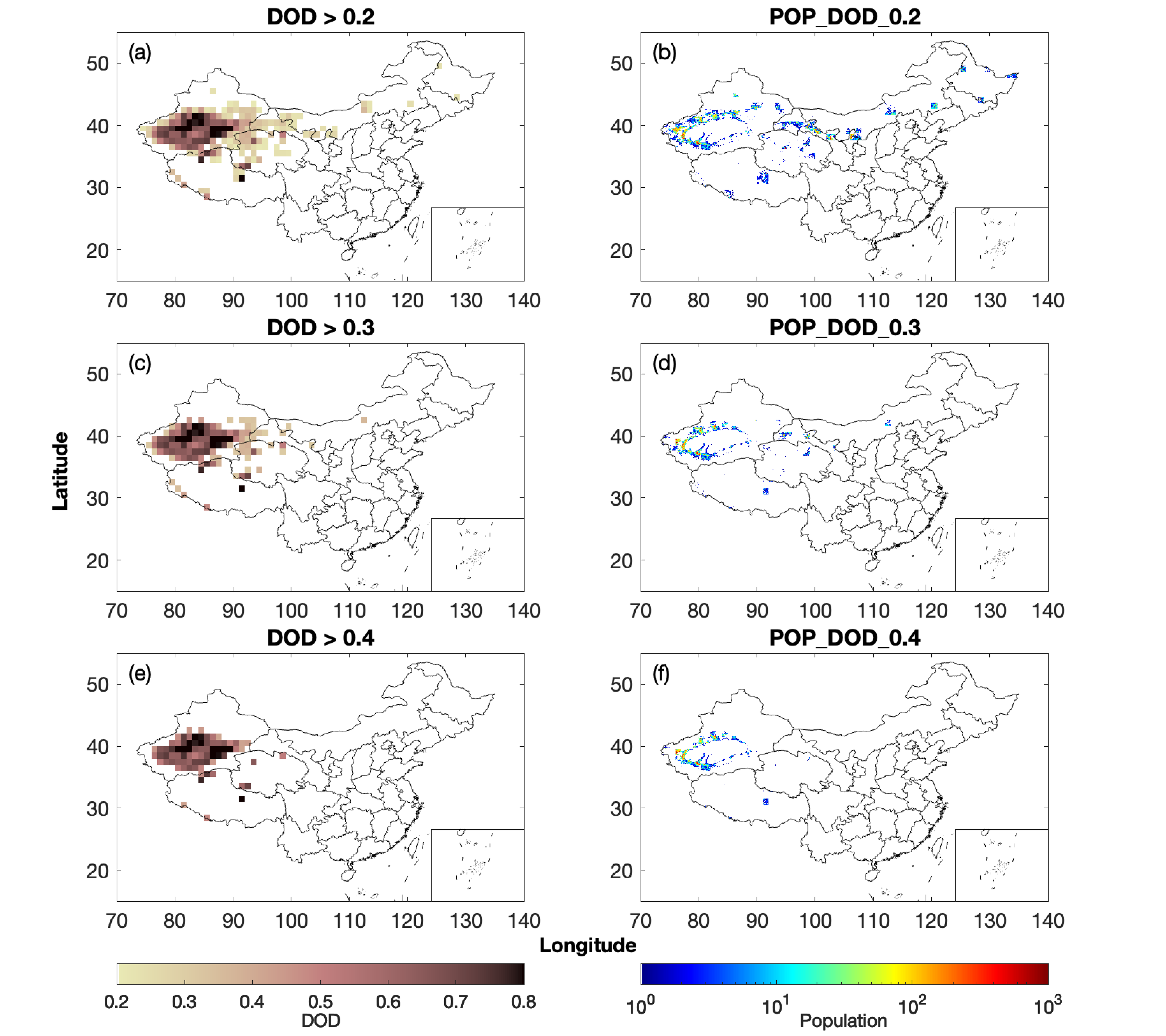

Supplement: S12 Fig — Locations with March-April mean DOD above (a) 0.2, (c) 0.3, and (e) 0.4 in 2015 using MODIS and the spatial distribution of the population affected by DOD above (b) 0.2, (d) 0.3, and (f) 0.4 in 2015. (TIF) [file pone.0281311.s012.tif]

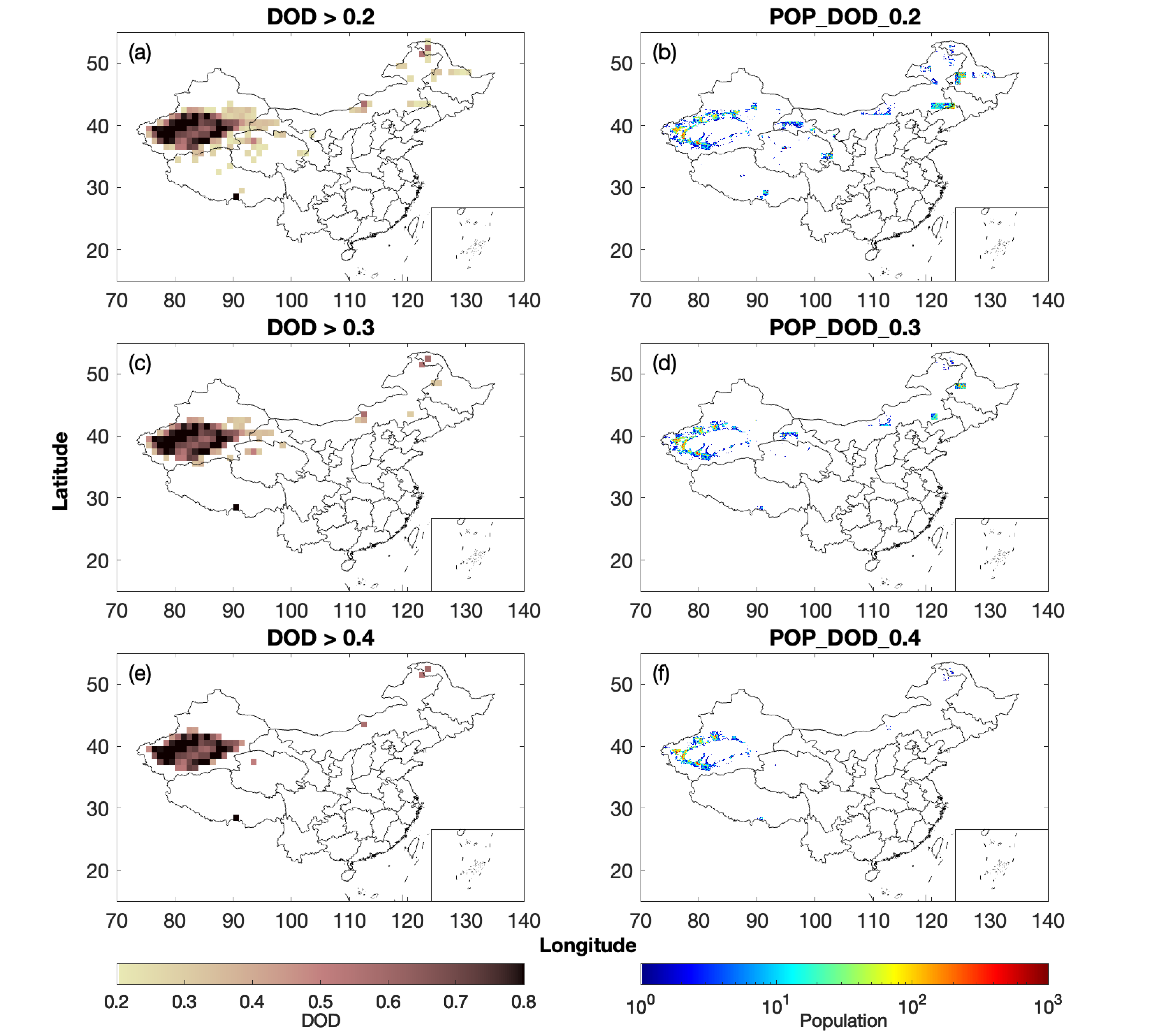

Supplement: S13 Fig — Locations with March-April mean DOD above (a) 0.2, (c) 0.3, and (e) 0.4 in 2016 using MODIS and the spatial distribution of the population affected by DOD above (b) 0.2, (d) 0.3, and (f) 0.4 in 2016. (TIF) [file pone.0281311.s013.tif]

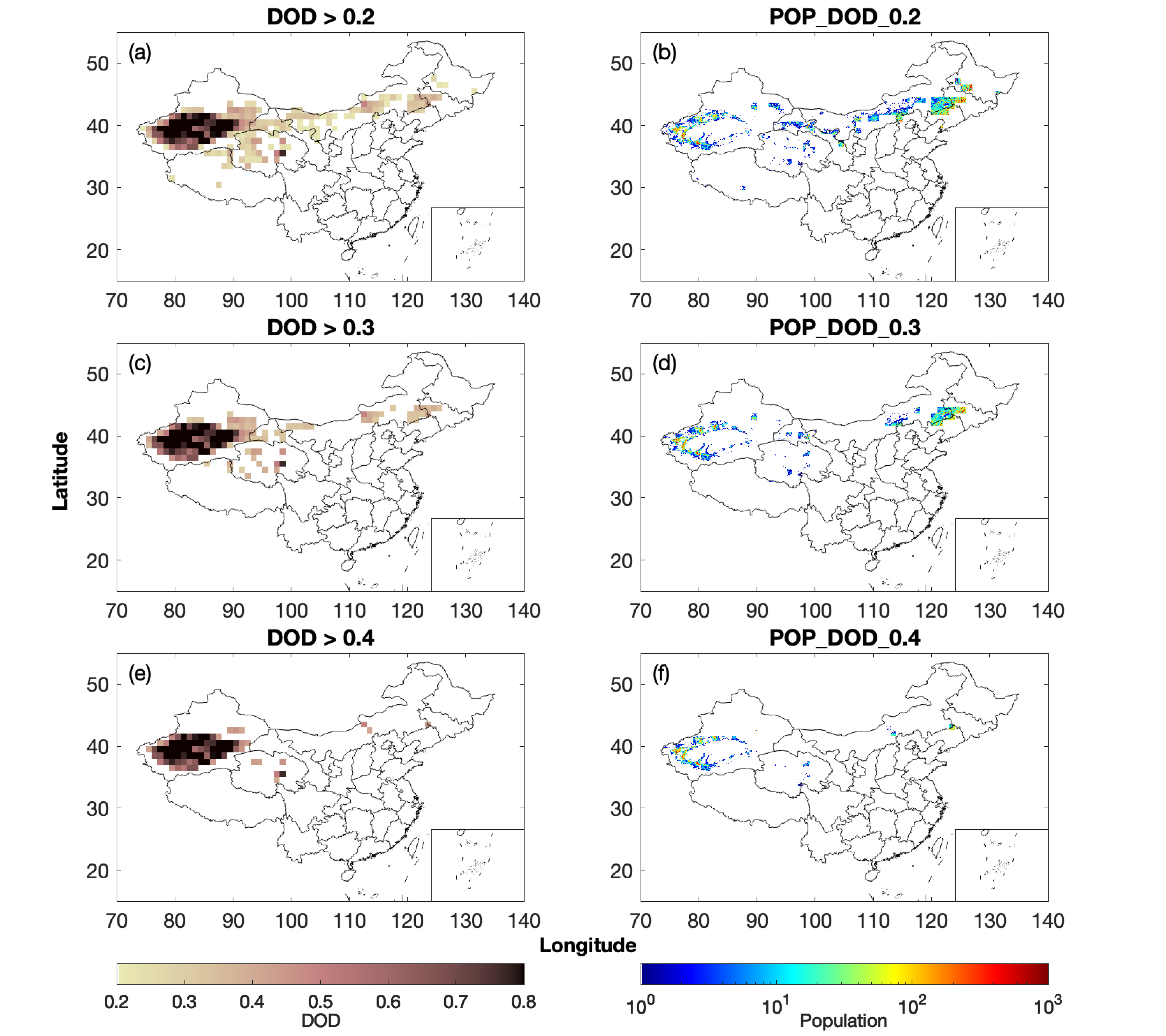

Supplement: S14 Fig — Locations with March-April mean DOD above (a) 0.2, (c) 0.3, and (e) 0.4 in 2018 using MODIS and the spatial distribution of the population affected by DOD above (b) 0.2, (d) 0.3, and (f) 0.4 in 2018. (TIF) [file pone.0281311.s014.tif]

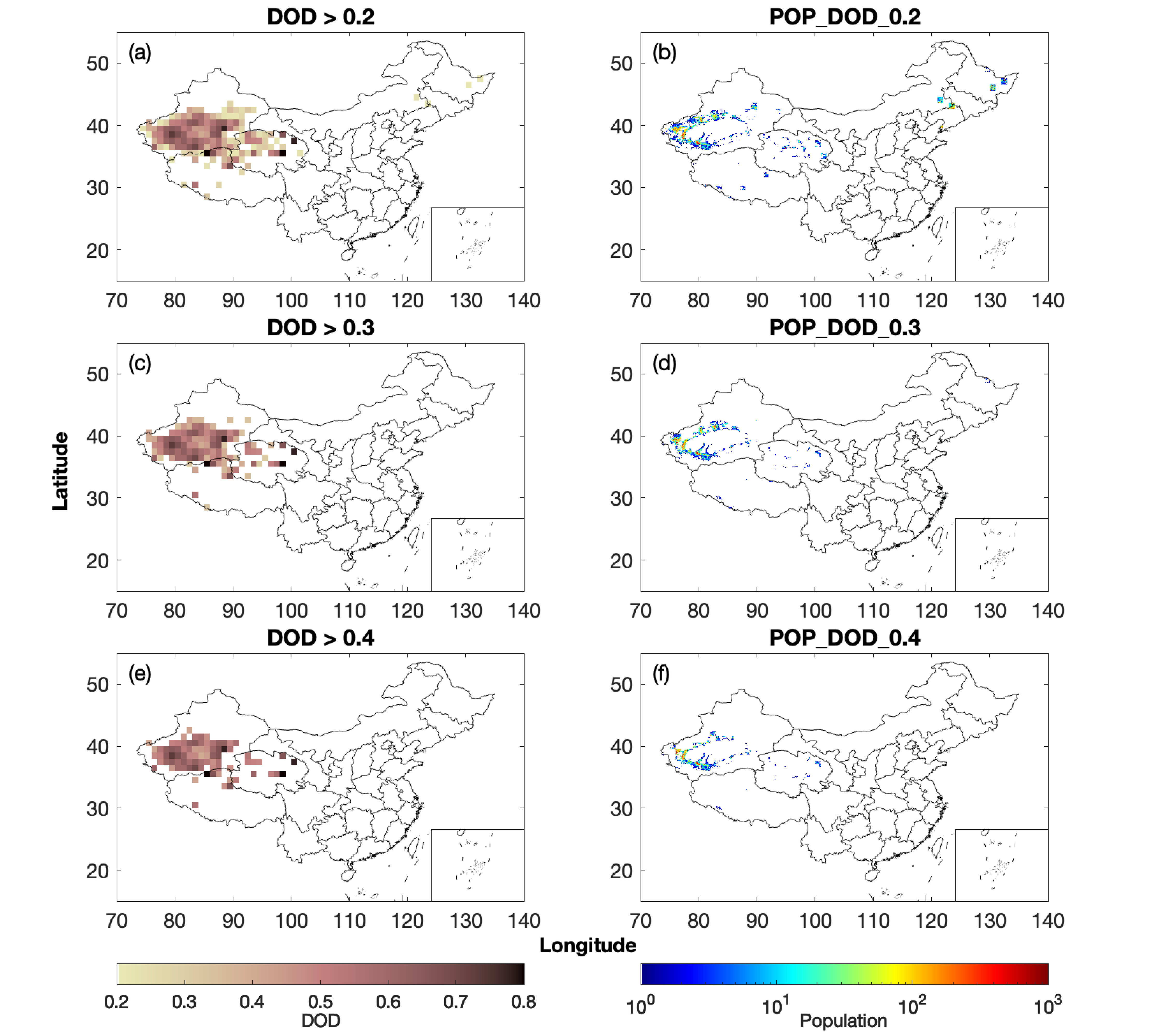

Supplement: S15 Fig — Locations with March-April mean DOD above (a) 0.2, (c) 0.3, and (e) 0.4 in 2019 using MODIS and the spatial distribution of the population affected by DOD above (b) 0.2, (d) 0.3, and (f) 0.4 in 2019. (TIF) [file pone.0281311.s015.tif]

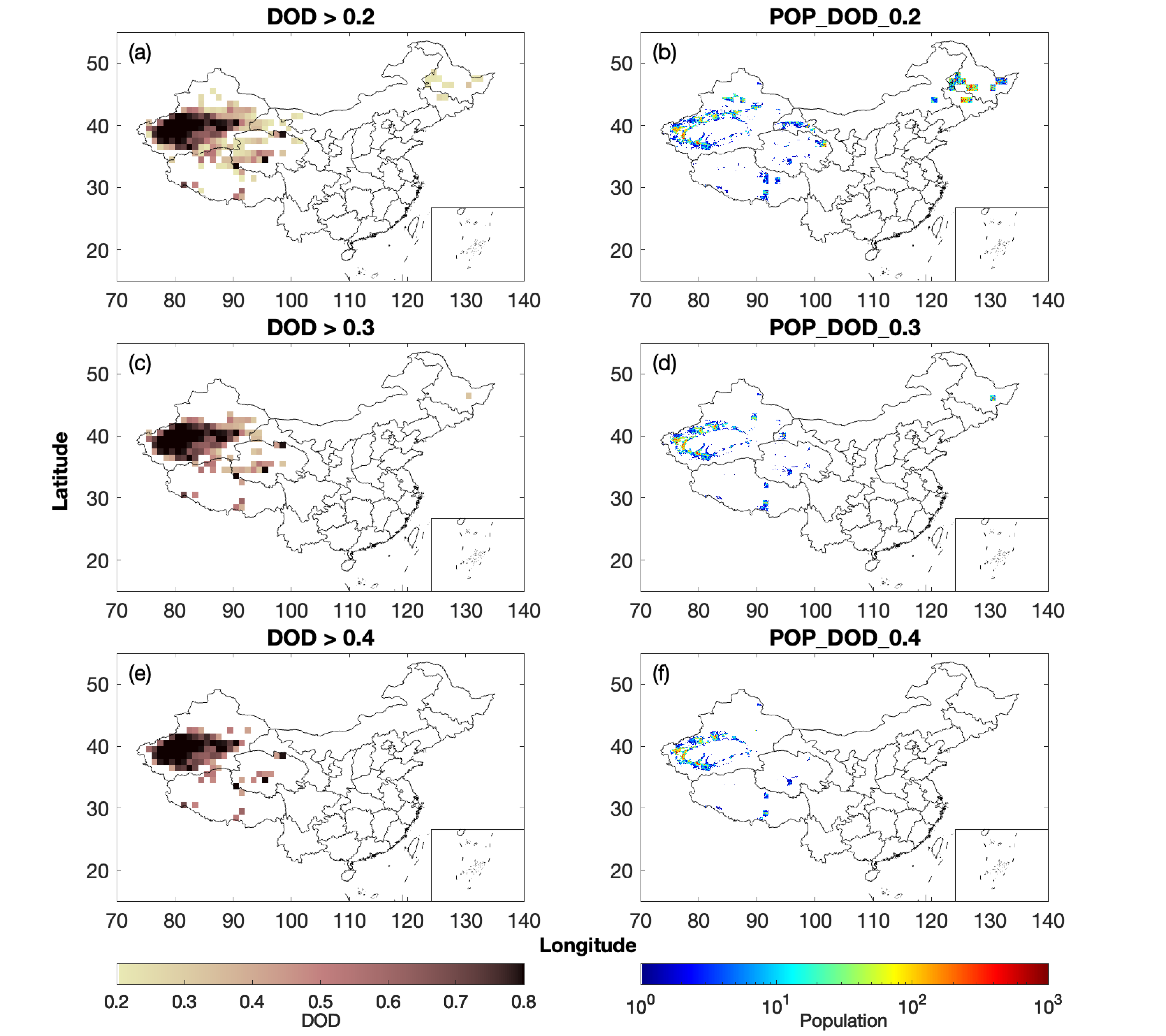

Supplement: S16 Fig — Locations with March-April mean DOD above (a) 0.2, (c) 0.3, and (e) 0.4 in 2020 using MODIS and the spatial distribution of the population affected by DOD above (b) 0.2, (d) 0.3, and (f) 0.4 in 2020. (TIF) [file pone.0281311.s016.tif]

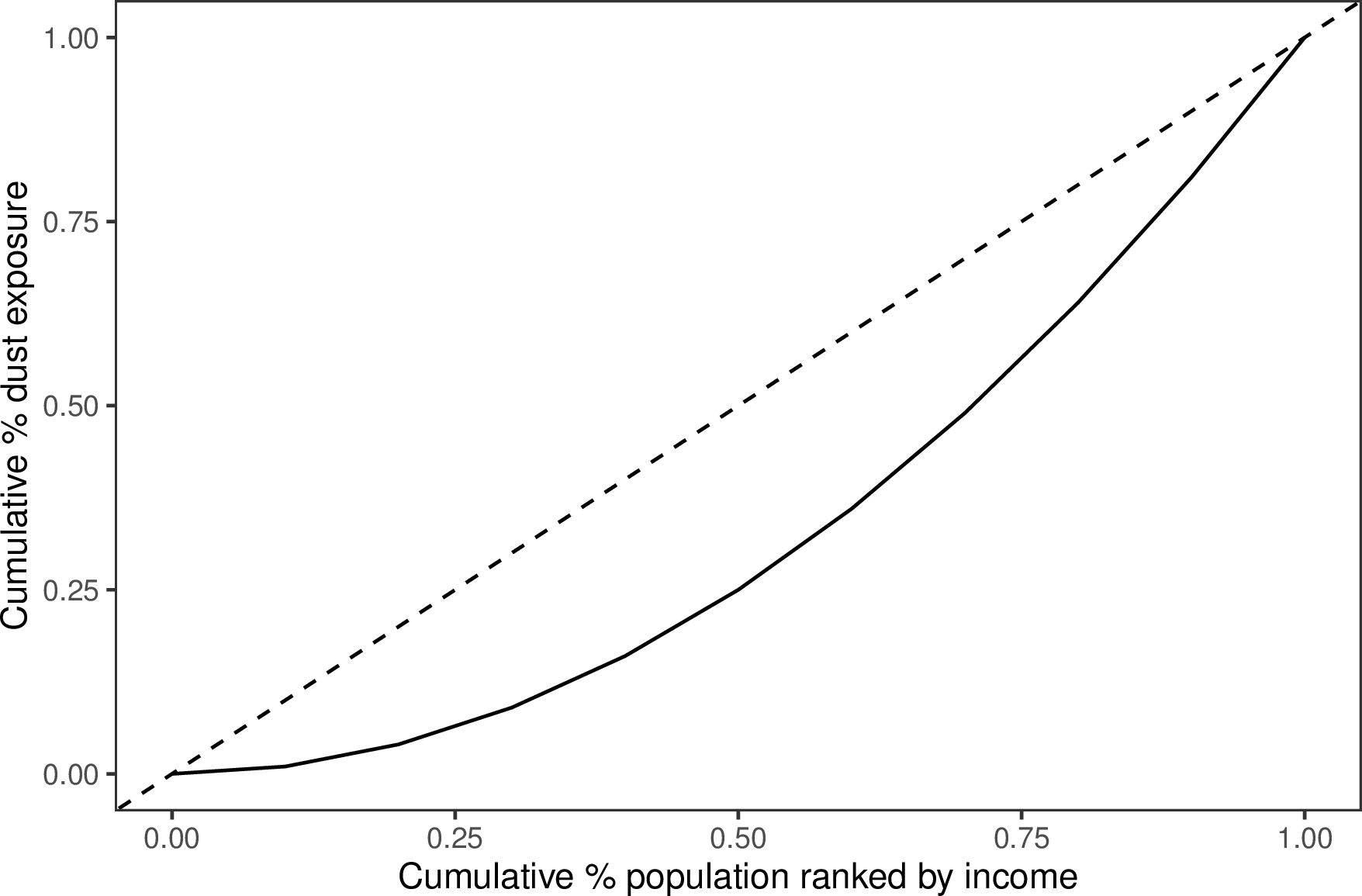

Supplement: S17 Fig — (TIF) [file pone.0281311.s017.tif]
