# Supplementary material for: The population affected by dust in China in the springtime
Source: PLoS One. 2024 Feb 23;19(2):e0281311. doi: 10.1371/journal.pone.0281311 (PMC10889670; doi:10.1371/journal.pone.0281311)
Supplement: S1 Table — (DOCX) [file pone.0281311.s018.docx]

S1. The population (in million) affected by March-April mean DOD above 0.2 in each province from 2003 to 2020.

|  | Provinces | 2003 | 2004 | 2005 | 2006 | 2007 | 2008 | 2009 | 2010 | 2011 | 2012 | 2013 | 2014 | 2015 | 2016 | 2017 | 2018 | 2019 | 2020 |
| --- | --- | --- | --- | --- | --- | --- | --- | --- | --- | --- | --- | --- | --- | --- | --- | --- | --- | --- | --- |
| NW | Xinjiang | 8.0 | 12.3 | 6.5 | 10.5 | 13.9 | 10.4 | 14.0 | 10.9 | 11.0 | 11.0 | 11.4 | 12.4 | 12.2 | 11.7 | 9.1 | 11.6 | 11.1 | 18.7 |
|  | Gansu | 16.9 | 15.1 | 2.5 | 21.8 | 16.3 | 4.4 | 9.0 | 24.0 | 18.6 | 6.8 | 24.1 | 5.3 | 3.7 | 1.0 | 3.1 | 2.5 | - | 1.5 |
|  | Qinghai | 2.6 | 1.4 | 4.2 | 4.8 | 5.0 | 0.9 | 2.7 | 5.2 | 2.7 | 0.9 | 4.6 | 2.9 | 0.5 | 0.5 | 1.2 | 0.3 | 0.7 | 2.6 |
|  | Ningxia | 5.5 | 4.9 | - | 5.9 | 5.9 | - | 5.2 | 6.3 | 2.8 | 4.6 | 5.7 | - | 3.0 | - | - | - | - | - |
|  | Shaanxi | 1.5 | 0.3 | - | 6.1 | 1.9 | - | 0.8 | 16.3 | 0.2 | - | 14.2 | - | - | - | - | - | - | - |
| N | Inner Mongolia | 2.1 | 4.6 | 4.6 | 15.8 | 3.1 | 5.2 | 4.7 | 15.8 | 9.4 | 4.6 | 0.5 | 0.2 | 1.1 | 2.3 | 1.4 | 8.8 | 0.6 | 0.5 |
|  | Beijing | - | - | - | 1.0 | - | - | - | - | - | - | - | - | - | - | - | - | - | - |
|  | Tianjin | - | - | - | 10.6 | - | - | - | - | - | - | - | - | - | - | - | - | - | - |
|  | Hebei | 0.8 | - | - | 34.9 | - | - | - | 7.4 | 0.3 | - | - | - | - | - | - | 1.8 | - | - |
|  | Shanxi | 0.4 | - | - | 3.7 | - | - | - | 14.0 | - | - | 0.7 | - | - | - | - | - | - | - |
| NE | Heilongjiang | - | 0.3 | 1.4 | 12.3 | 0.2 | - | - | 7.4 | 0.6 | 0.1 | 2.4 | - | 0.7 | 3.5 | 1.2 | 10.5 | 1.9 | 16.0 |
|  | Liaoning | - | - | 3.5 | 26.0 | 1.8 | - | 0.1 | 1.6 | 0.8 | - | 0.8 | - | - | 0.8 | 0.1 | 5.3 | 1.3 | - |
|  | Jilin | - | - | 4.3 | 17.0 | 4.9 | - | - | 16.3 | 1.0 | - | - | - | - | 0.2 | - | 8.0 | 0.2 | 9.2 |
| SW | Sichuan | - | - | - | - | - | - | 11.6 | - | 0.1 | - | - | - | - | - | - | - | - | - |
|  | Tibet | - | - | - | 0.1 | 0.2 | 0.1 | 0.2 | 0.4 | 0.1 | - | - | 0.1 | 0.2 | 0.2 | - | - | 0.1 | 0.4 |
| E | Henan | - | - | - | - | - | - | - | 4.3 | - | - | - | - | - | - | - | - | - | - |
|  | Shandong | 0.3 | - | 0.3 | 37.8 | 0.3 | - | - | 1.8 | 0.3 | 0.3 | - | - | - | - | - | 0.2 | - | - |
|  | Jiangsu | - | - | - | 0.1 | - | - | - | 9.5 | - | - | - | - | - | - | - | - | - | - |
|  | Anhui | - | - | - | - | - | - | - | 1.0 | - | - | - | - | - | - | - | - | - | - |
|  | Total | 38.1 | 38.9 | 27.3 | 208.4 | 53.5 | 21.0 | 48.3 | 142.2 | 47.9 | 28.3 | 64.4 | 20.9 | 21.4 | 20.2 | 16.1 | 49.0 | 15.9 | 48.9 |
