# Supplementary material for: The population affected by dust in China in the springtime
Source: PLoS One. 2024 Feb 23;19(2):e0281311. doi: 10.1371/journal.pone.0281311 (PMC10889670; doi:10.1371/journal.pone.0281311)
Supplement: S2 Table — (DOCX) [file pone.0281311.s019.docx]

S2. The population (in million) affected by March-April mean DOD > 0.3 in each province from 2003 to 2020.

|  | Provinces | 2003 | 2004 | 2005 | 2006 | 2007 | 2008 | 2009 | 2010 | 2011 | 2012 | 2013 | 2014 | 2015 | 2016 | 2017 | 2018 | 2019 | 2020 |
| --- | --- | --- | --- | --- | --- | --- | --- | --- | --- | --- | --- | --- | --- | --- | --- | --- | --- | --- | --- |
| NW | Xinjiang | 7.7 | 7.2 | 5.2 | 10.4 | 9.4 | 9.1 | 9.3 | 9.8 | 9.9 | 9.6 | 9.4 | 10.9 | 8.6 | 10.1 | 8.3 | 10.8 | 9.9 | 11.4 |
|  | Gansu | 9.6 | 4.1 | 0.3 | 15.9 | 10.5 | 2.7 | 4.9 | 18.3 | 5.6 | 4.3 | 17.3 | 0.8 | 1.1 | 0.4 | 1.4 | 1.0 | - | 0.2 |
|  | Qinghai | 1.9 | 0.1 | 2.3 | 4.4 | 3.7 | 0.3 | 2.3 | 4.7 | 1.2 | 0.2 | 3.5 | 0.2 | 0.1 | - | 0.4 | 0.1 | 0.1 | 0.1 |
|  | Ningxia | 1.4 | 1.4 | - | 5.8 | 5.1 | - | 1.2 | 6.3 | 0.6 | 1.0 | 2.8 | - | - | - | - | - | - | - |
|  | Shaanxi | - | - | - | 3.8 | 1.8 | - | 0.2 | 5.0 | - | - | - | - | - | - | - | - | - | - |
| N | Inner Mongolia | - | 0.4 | 2.7 | 8.7 | 0.3 | - | - | 9.1 | 1.1 | 0.5 | - | - | - | 0.7 | - | 4.0 | - | - |
|  | Hebei | - | - | - | 0.2 | - | - | - | 0.1 | 0.2 | - | - | - | - | - | - | 0.2 | - | - |
|  | Shanxi | - | - | - | 0.5 | - | - | - | 4.4 | - | - | - | - | - | - | - | - | - | - |
| NE | Heilongjiang | - | - | - | 1.4 | - | - | - | 5.1 | - | - | 0.5 | - | - | 1.8 | 0.2 | - | - | 1.5 |
|  | Liaoning | - | - | 0.8 | 6.4 | - | - | - | 0.1 | - | - | - | - | - | - | - | 3.2 | - | - |
|  | Jilin | - | - | - | 3.6 | 1.0 | - | - | 3.2 | - | - | - | - | - | - | - | 8.0 | - | - |
| SW | Sichuan | - | - | - | - | - | - | - | - | 0.1 | - | - | - | - | - | - | - | - | - |
|  | Tibet | - | - | - | - | 0.1 | - | - | 0.3 | - | - | - | 0.1 | - | - | - | - | - | 0.3 |
| E | Henan | - | - | - | - | - | - | - | 4.3 | - | - | - | - | - | - | - | - | - | - |
|  | Shandong | - | - | - | 14.1 | - | - | - | - | 0.3 | 0.3 | - | - | - | - | - | - | - | - |
|  | Total | 20.6 | 13.2 | 11.3 | 75.2 | 31.9 | 12.1 | 17.9 | 70.7 | 19.0 | 15.9 | 33.5 | 12.0 | 9.8 | 13.0 | 10.3 | 27.3 | 10.0 | 13.5 |
