# Supplementary material for: The population affected by dust in China in the springtime
Source: PLoS One. 2024 Feb 23;19(2):e0281311. doi: 10.1371/journal.pone.0281311 (PMC10889670; doi:10.1371/journal.pone.0281311)
Supplement: S3 Table — (DOCX) [file pone.0281311.s020.docx]

S3. The population (in million) affected by March-April mean DAOD > 0.4 in each province from 2003 to 2020.

|  | Provinces | 2003 | 2004 | 2005 | 2006 | 2007 | 2008 | 2009 | 2010 | 2011 | 2012 | 2013 | 2014 | 2015 | 2016 | 2017 | 2018 | 2019 | 2020 |
| --- | --- | --- | --- | --- | --- | --- | --- | --- | --- | --- | --- | --- | --- | --- | --- | --- | --- | --- | --- |
| NW | Xinjiang | 5.9 | 5.8 | 3.1 | 10.1 | 9.4 | 8.0 | 7.3 | 9.4 | 8.6 | 8.3 | 8.2 | 8.7 | 8.1 | 9.5 | 3.8 | 9.8 | 7.0 | 10.7 |
|  | Gansu | 0.3 | - | - | 8.4 | 1.9 | - | 2.9 | 10.3 | - | 1.2 | 6.2 | - | - | - | - | - | - | - |
|  | Qinghai | 0.2 | - | 2.1 | 2.5 | 3.3 | 0.2 | 0.2 | 3.6 | 0.2 | - | 0.2 | - | - | - | 0.2 | 0.1 | 0.1 | 0.1 |
|  | Ningxia | - | - | - | 5.8 | 0.3 | - | - | 2.8 | - | - | - | - | - | - | - | - | - | - |
|  | Shaanxi | - | - | - | 3.2 | - | - | - | 2.9 | - | - | - | - | - | - | - | - | - | - |
| N | Inner Mongolia | - | 0.1 | 1.3 | 2.4 | - | - | - | 2.3 | 0.3 | - | - | - | - | 0.1 | - | 0.4 | - | - |
|  | Hebei | - | - | - | - | - | - | - | - | 0.2 | - | - | - | - | - | - | - | - | - |
|  | Shanxi | - | - | - | 0.5 | - | - | - | - | - | - | - | - | - | - | - | - | - | - |
| NE | Heilongjiang | - | - | - | - | - | - | - | 1.5 | - | - | - | - | - | - | - | - | - | - |
|  | Liaoning | - | - | - | 0.6 | - | - | - | - | - | - | - | - | - | - | - | 0.8 | - | - |
|  | Jilin | - | - | - | 1.6 | - | - | - | 0.5 | - | - | - | - | - | - | - | 0.2 | - | - |
| SW | Sichuan | - | - | - | - | - | - | - | - | 0.1 | - | - | - | - | - | - | - | - | - |
|  | Tibet | - | - | - | 0.1 | 0.1 | - | - | 0.3 | - | - | - | 0.1 | 0.1 | - | - | - | - | 0.2 |
| E | Shandong | - | - | - | 0.3 | - | - | - | - | 0.3 | 0.3 | - | - | - | - | - | - | - | - |
|  | Total | 6.4 | 5.9 | 6.5 | 35.5 | 15 | 8.2 | 10.4 | 33.6 | 9.7 | 9.8 | 14.6 | 8.8 | 8.2 | 9.6 | 4.0 | 11.3 | 7.1 | 11.0 |
