# Supplementary material for: The population affected by dust in China in the springtime
Source: PLoS One. 2024 Feb 23;19(2):e0281311. doi: 10.1371/journal.pone.0281311 (PMC10889670; doi:10.1371/journal.pone.0281311)
Supplement: Attachment — Submitted filename: Response to Reviewers.docx [file pone.0281311.s021.docx]

**Reviewer #1:**

We would like to thank the reviewer for these helpful comments.

I found this topic to be extremely interesting, and I was very impressed with the way in which the authors presented their findings. Following are a few minor observations and comments that may help to further enhance your research work. The authors should address the following points in detail.

1. The abstract should discuss the paper achievement.

We have discussed the paper's achievement in the abstract.

2. Introduction of paper should include the contribution more prominently.

We have talked about the contribution in L58-66.

3. A “Literature Review” section/subsection should be added in the paper. The literature
review section should include a tabular comparison of existing models to identify their
shortcomings and strengths. The literature section is lacking in enclosure of formulation and
pollution aspect and requires more recent studies to be reviewed, therefore I suggest you cite 2022 and 2021 papers including the following works:
[https://doi.org/10.1016/j.chemosphere.2021.132569](https://urldefense.com/v3/__https:/doi.org/10.1016/j.chemosphere.2021.132569__;!!OToaGQ!vFMMx-SFyJUddv2rA2QYvHB5slm_knOMRZFFnlDRjRQJkSmdOxkgZ-oYS54l9CnX8cSCUSLnjEQtXHcZsgkaXA$)
[https://doi.org/10.1155/2022/6430120](https://urldefense.com/v3/__https:/doi.org/10.1155/2022/6430120__;!!OToaGQ!vFMMx-SFyJUddv2rA2QYvHB5slm_knOMRZFFnlDRjRQJkSmdOxkgZ-oYS54l9CnX8cSCUSLnjEQtXHdqqugYBQ$)
[http://dx.doi.org/10.36785/jaes.111501](https://urldefense.com/v3/__http:/dx.doi.org/10.36785/jaes.111501__;!!OToaGQ!vFMMx-SFyJUddv2rA2QYvHB5slm_knOMRZFFnlDRjRQJkSmdOxkgZ-oYS54l9CnX8cSCUSLnjEQtXHeEAm6oVg$)
[https://doi.org/10.15244/pjoes/148065](https://urldefense.com/v3/__https:/doi.org/10.15244/pjoes/148065__;!!OToaGQ!vFMMx-SFyJUddv2rA2QYvHB5slm_knOMRZFFnlDRjRQJkSmdOxkgZ-oYS54l9CnX8cSCUSLnjEQtXHetagxkNg$)
[https://doi.org/10.3389/fenvs.2022.945628](https://urldefense.com/v3/__https:/doi.org/10.3389/fenvs.2022.945628__;!!OToaGQ!vFMMx-SFyJUddv2rA2QYvHB5slm_knOMRZFFnlDRjRQJkSmdOxkgZ-oYS54l9CnX8cSCUSLnjEQtXHc8_JJWNA$)

Thank you for the suggestions. We have cited the most relevant papers in the revised manuscript. Since the manuscript guideline of PLOS ONE does not include a literature review section, we have included these papers in introduction (L45-47, reference 29-31). To our best knowledge, this is the first work estimating the population affected by dust in China, and thus there are no other works to compare with. However, we have compared our findings with previous works on air pollution in discussion (section 4.2, L310-315).

4. Define the preliminary in the methodology section to explain all the mathematical notations and symbols used in the paper.

All the mathematical notations and symbols have been explained in section 2.3.

5. It is necessary to improve the quality of the figures.

The quality of the figures has been improved.

6. The authors should consider the comparison of results with existing studies.

We have compared our results with previous findings (section 4.2, L310-315, section 4.3, L329-340).

7. The authors should consider serious revision of formatting.

The formatting of the manuscript is now in accordance with the guideline of PLOS ONE.

**Reviewer #2:** 

We would like to thank the reviewer for these helpful comments.

The present study we design is to find out the socioeconomic impacts of dust storms in China. I appreciate the general set-up of the experiment, but I also outline concerns below in the detailed comments that need attention before the study can be published.

1. Please don’t put title words in keywords.

Keywords have been removed.

2. Please provide policy recommendations on how to prevent these populations from the dust.

We have provided policy recommendations in introduction (L58-66) and discussion (section 4.3, L332-340).

3. Introduction
Please increase the introduction to the following related literature.
[https://www.sciencedirect.com/science/article/pii/S0045653521030411](https://urldefense.com/v3/__https:/www.sciencedirect.com/science/article/pii/S0045653521030411__;!!OToaGQ!vFMMx-SFyJUddv2rA2QYvHB5slm_knOMRZFFnlDRjRQJkSmdOxkgZ-oYS54l9CnX8cSCUSLnjEQtXHcu_d4uNQ$)
[https://www.mdpi.com/2073-4433/12/10/1338](https://urldefense.com/v3/__https:/www.mdpi.com/2073-4433/12/10/1338__;!!OToaGQ!vFMMx-SFyJUddv2rA2QYvHB5slm_knOMRZFFnlDRjRQJkSmdOxkgZ-oYS54l9CnX8cSCUSLnjEQtXHeohZZ_ng$)

Thank you for the suggestion. We have included these two papers in introduction (L45-47, reference 29,30).

4. Discussion
Better add similar heading as you put in the result. The discussion part needs to improve by using previous studies. Please start each paragraph with your finding and then a possible reason for this finding and compare it with previous research. I think plenty of literature is available in this regard. You can also use air pollution research in this regard. The discussion part has only two references. Please include more references and enrich your discussion.

Headings have been added to discussion. We have included more references in discussion and compared our results with previous findings (L310-315, L329-340).

5. Please add the implication of your study.

We have added the implication in introduction (L58-66).
