# Supplementary material for: The population affected by dust in China in the springtime
Source: PLoS One. 2024 Feb 23;19(2):e0281311. doi: 10.1371/journal.pone.0281311 (PMC10889670; doi:10.1371/journal.pone.0281311)
Supplement: Attachment — Submitted filename: Response to Reviewers.docx [file pone.0281311.s022.docx]

**Reviewer #1:**

We would like to thank the reviewer for these helpful comments.

I found this topic to be extremely interesting, and I was very impressed with the way in which the authors presented their findings. Following are a few minor observations and comments that may help to further enhance your research work. The authors should address the following points in detail.

1. The abstract should discuss the paper achievement.

We have discussed the paper's achievement in the abstract.

2. Introduction of paper should include the contribution more prominently.

We have talked about the contribution in L58-66.

3. A “Literature Review” section/subsection should be added in the paper. The literature
review section should include a tabular comparison of existing models to identify their
shortcomings and strengths. The literature section is lacking in enclosure of formulation and
pollution aspect and requires more recent studies to be reviewed, therefore I suggest you cite 2022 and 2021 papers including the following works:
[https://doi.org/10.1016/j.chemosphere.2021.132569](https://urldefense.com/v3/__https:/doi.org/10.1016/j.chemosphere.2021.132569__;!!OToaGQ!vFMMx-SFyJUddv2rA2QYvHB5slm_knOMRZFFnlDRjRQJkSmdOxkgZ-oYS54l9CnX8cSCUSLnjEQtXHcZsgkaXA$)
[https://doi.org/10.1155/2022/6430120](https://urldefense.com/v3/__https:/doi.org/10.1155/2022/6430120__;!!OToaGQ!vFMMx-SFyJUddv2rA2QYvHB5slm_knOMRZFFnlDRjRQJkSmdOxkgZ-oYS54l9CnX8cSCUSLnjEQtXHdqqugYBQ$)
[http://dx.doi.org/10.36785/jaes.111501](https://urldefense.com/v3/__http:/dx.doi.org/10.36785/jaes.111501__;!!OToaGQ!vFMMx-SFyJUddv2rA2QYvHB5slm_knOMRZFFnlDRjRQJkSmdOxkgZ-oYS54l9CnX8cSCUSLnjEQtXHeEAm6oVg$)
[https://doi.org/10.15244/pjoes/148065](https://urldefense.com/v3/__https:/doi.org/10.15244/pjoes/148065__;!!OToaGQ!vFMMx-SFyJUddv2rA2QYvHB5slm_knOMRZFFnlDRjRQJkSmdOxkgZ-oYS54l9CnX8cSCUSLnjEQtXHetagxkNg$)
[https://doi.org/10.3389/fenvs.2022.945628](https://urldefense.com/v3/__https:/doi.org/10.3389/fenvs.2022.945628__;!!OToaGQ!vFMMx-SFyJUddv2rA2QYvHB5slm_knOMRZFFnlDRjRQJkSmdOxkgZ-oYS54l9CnX8cSCUSLnjEQtXHc8_JJWNA$)

Thank you for the suggestions. We have cited the most relevant papers in the revised manuscript. Since the manuscript guideline of PLOS ONE does not include a literature review section, we have included these papers in introduction (L45-47, reference 29-31). To our best knowledge, this is the first work estimating the population affected by dust in China, and thus there are no other works to compare with. However, we have compared our findings with previous works on air pollution in discussion (section 4.2, L310-315).

4. Define the preliminary in the methodology section to explain all the mathematical notations and symbols used in the paper.

All the mathematical notations and symbols have been explained in section 2.3.

5. It is necessary to improve the quality of the figures.

The quality of the figures has been improved.

6. The authors should consider the comparison of results with existing studies.

We have compared our results with previous findings (section 4.2, L310-315, section 4.3, L329-340).

7. The authors should consider serious revision of formatting.

The formatting of the manuscript is now in accordance with the guideline of PLOS ONE.

**Reviewer #2:** 

We would like to thank the reviewer for these helpful comments.

The present study we design is to find out the socioeconomic impacts of dust storms in China. I appreciate the general set-up of the experiment, but I also outline concerns below in the detailed comments that need attention before the study can be published.

1. Please don’t put title words in keywords.

Keywords have been removed.

2. Please provide policy recommendations on how to prevent these populations from the dust.

We have provided policy recommendations in introduction (L58-66) and discussion (section 4.3, L332-340).

3. Introduction
Please increase the introduction to the following related literature.
[https://www.sciencedirect.com/science/article/pii/S0045653521030411](https://urldefense.com/v3/__https:/www.sciencedirect.com/science/article/pii/S0045653521030411__;!!OToaGQ!vFMMx-SFyJUddv2rA2QYvHB5slm_knOMRZFFnlDRjRQJkSmdOxkgZ-oYS54l9CnX8cSCUSLnjEQtXHcu_d4uNQ$)
[https://www.mdpi.com/2073-4433/12/10/1338](https://urldefense.com/v3/__https:/www.mdpi.com/2073-4433/12/10/1338__;!!OToaGQ!vFMMx-SFyJUddv2rA2QYvHB5slm_knOMRZFFnlDRjRQJkSmdOxkgZ-oYS54l9CnX8cSCUSLnjEQtXHeohZZ_ng$)

Thank you for the suggestion. We have included these two papers in introduction (L45-47, reference 29,30).

4. Discussion
Better add similar heading as you put in the result. The discussion part needs to improve by using previous studies. Please start each paragraph with your finding and then a possible reason for this finding and compare it with previous research. I think plenty of literature is available in this regard. You can also use air pollution research in this regard. The discussion part has only two references. Please include more references and enrich your discussion.

Headings have been added to discussion. We have included more references in discussion and compared our results with previous findings (L310-315, L329-340).

5. Please add the implication of your study.

We have added the implication in introduction (L58-66).

**Reviewer #3:** 

This paper, a revision of a manuscript I didn’t initially review, presents a really nicely-delivered intersection of two large datasets (the MODIS dust product, and population and socioeconomic data) over nearly two decades, and yields useful conclusions on the degree of human exposure to dust hazards over China.

We would like to thank the reviewer for these helpful comments. We have revised the manuscript accordingly.

Previous reviewers’ comments seem to have been well addressed in this revision, and I recommend its publication after a few relatively minor comments are addressed:

L35 Delete ‘Still’ – it does not follow logically

It has been deleted.

L43 “A previous study…”

The sentence has been revised (line 44).

L62-66 I appreciate that this section was added in direct response to a comment raised by both reviewers, but for me, it sits awkwardly here in the way it is worded. It reads, effectively as a set of conclusions at the end of the introduction, and comes immediately after the rationale for the study, which works very well in lines 58-62. Can these lines be altered to set these more as goals or ideas to be explored rather than as a fait accompli.

We have revised this section (lines 62-65).

L70 Suggest “MODIS (Moderate Resolution Imaging Spectroradiometer) instruments are aboard the Terra and Aqua satellites and..”

The sentence has been revised (line 69).

L114 and Figure 1 We are told here that the mean DOD in Xinjiang is 0.36 over the full time series, but it seems to me equally relevant to note that in southern half of Xinjiang in the Taklamakan it is more like >0.6. Averaging at a province level here seems to me to miss the point rather.

Considering this study is conducted on the provincial level, we use mean DOD over the entire province. It is true that DOD is highest over the Taklamakan. We have mentioned this in line 126.

L144-145 Worth noting here than 2004 is the prime example here?

Right. We have added lines 159-161.

L181 Refer to Fig. 1a at mention of the Heihe-Tengching line

Fig. 1a has been referred to (line 197).

L251-252 Specific mention of ‘visual inspection’ being inconclusive suggests to the reader than something else was/could be done to further explore this. Reword, or be more definitive that there is no clear relationship. Also, mention here of dust “generating” inequalities suggests a definite causal relationship. Might it not be that dust is generated in/affects rural areas more, which are poorer; this would be a correlation, not a causation.

We have revised this part (lines 267-268, 283).

L313 ‘pullulation’ = “population”, I assume?

Right. Corrected.

L350 “on the order of millions”

Corrected.

L353 “it is crucial to understand the future trend” – this has not been done in this study, and in fact you’ve just spend about eight lines explaining why this would be very difficult/impossible to do accurately. Why then just state that this is crucial?

The current understanding is that dust emission in East Asia would reduce in the 21^st^ century, but the magnitude is unclear. It is true that the future trend of dust is beyond the scope of this work, but it would be something to look at for future research. These eight lines might be redundant, so we removed a few lines. The point here is to say that although the population affected by dust is likely to decrease, it is still necessary to study such topic, and this research will be carried on as more data become available.

**Reviewer #4:**

We would like to thank the reviewer for these helpful comments. We have revised the manuscript accordingly.

This study presents the spatiotemporal variations of population affected by dust in China and investigates the inequalities in dust impacts on different socioeconomic groups. To my best knowledge, there are not many studies on this topic, although the dust-affected regions and population are reported for some dust events. Most of the manuscript is clearly presented and well written. I have some comments for further consideration.

Major comments;
Section 4.1, in particular “more useful” in Line 289: Although I agree there are some advantages in DOD compared to surface records, there are at least two caveats in DOD for assessing the impacts of dust-affected population. First, although monthly DOD is provided, this does not represent the real distribution of dust, as some dust distributions cannot be distinguished by satellites due to the cloud cover and they are often represented as missing data. Second, DOD represents the dust in the total column, while humans are only exposed to the dust particles near the surface. There is some relationship between surface dust concentration and DOD, but there is some uncertainty. Although this work is useful and a good reference for the community, the authors should not overstate their work and should be aware of its caveats.

It is true that satellite observations also have caveats. We have discussed this point in lines 313-318.

Lines 120-121: Description of dust emission and transportation is not accurate: Please be aware that dust particles in Taklimakan Deserts are mostly trapped within the Tarim Basin, while the dust originating from Gobi Deserts is mainly transported to downwind regions including North China and East China.

We have added lines 131-133 to be accurate.

Lines 123-124: Not only dust from eastern Inner Mongolia but also dust from Gobi Deserts.

The sentence has been revised (line 139).

Lines 336-338: This sentence is misleading. Similar forecast models for predicting East Asia dust are already available to provide the forecast for the public.

The sentence has been removed.

Specific comments:
Line 43: The paragraphs are not well edited and it is a bit difficult to find the beginning of the paragraph. One blank row is needed between this paragraph and the previous paragraph.

One blank row has been added.

Line 88, Section 2.3: To facilitate the explanation, I suggest the authors provides the figure in accompany with the equation for description (probably in supplemental file).

We have included a concentration curve in the supplemental appendix to illustrate the equation.

Line 118: central China -> North China?

Shanxi and Hebei are located in central China.

Line 127: It is difficult to find the orders of figure in the appendix.

The figures are listed by year from 2003 through 2020 (except for 2006 and 2017, which are in the main text) in the appendix.

Lines 131-132: I cannot find the reference. I think it may be the Annual Book of Meteorological Disasters in China for 2018 (2019), as the records ended in 2018.

The number of dust events are from the Annual Book of Meteorological Disasters in China (2019). We have added the reference (reference no XX).

Lines 199-200: the Municipality of Tianjin -> Tianjin?

Revised (line 225).

Line 284: meteorological factors: It is not clear to me.

The sentence has been revised (line 333).

Line 314: 142 million: I think the number cannot be directly compared to that in Liu et al. (2007), as different criteria are used between this study and that study.

One of the reviewers in the first round suggested we compare this study with other studies. However, we were unable to find other studies that estimated the population affected by dust of the entire country, and thus we compared our study with studies that estimated the population affected by air pollution since dust is particulate matter, and particulate matter is considered as a pollutant. We agree with the reviewer that it is not a fair comparison as the criteria used are different. But in order to respond to the comment raised by one of the reviewers, we added this comparison. We have added one sentence to indicate that the criteria used are different (lines 371-372).

Lines 323-325: Inequalities indicate the difference between poor and wealthy peoples, but they do not imply that healthcare does not improve in past decades. Please clarify there is not missing leading information.

The study conducted by Song et al. (2019) shows that although health resources (health professionals and hospital beds per 1000) increased in rural China after the health care reform in 2009, the differences in health resource between the wealthiest and poorest counties were enlarging (lines 389-391).

Song S, Yuan B, Zhang L, Cheng G, Zhu W, Hou Z, et al. Increased inequalities in health resource and access to health care in rural China. International Journal of Environmental Research and Public Health. 2019;16:49.

Lines 367-369: people … affected: It is not clear to me.

We have revised the sentence (lines 446-448).

Line 370: "risk management" is not clear to me.

The sentence has been revised (line 456).
